# Socioeconomic influence on treatment and outcome of patients with oral cancer in Germany

**DOI:** 10.1007/s10006-021-00997-1

**Published:** 2021-08-26

**Authors:** Christoph Klingelhöffer, Annegret Obst, Johannes K. Meier, Torsten E. Reichert, Tobias Ettl, Steffen Mueller

**Affiliations:** Department of Cranio- and Maxillofacial Surgery, University Hospital Regensburg, Franz-Josef-Strauß-Allee 11, 93053 Regensburg, Germany

**Keywords:** Oral cancer, Survival, Socioeconomic factors, Marriage, Health insurance, Occupation

## Abstract

**Purpose:**

To illustrate the influence of different socioeconomic factors on the treatment and outcome of patients in Germany with oral cancer.

**Methods:**

In this retrospective single-center study, 400 patients of our department of oral and maxillofacial surgery with primary cases of oral cancer were included. Preoperative diagnostics, occupational groups, and marital and health insurance status were evaluated. Overall and disease-specific survival were analyzed. Occupations were distinguished in 5 groups (unemployed, physically light workers, physically hard worker, university graduate, and freelancer). Data were adjusted to covariables like tumor size, positive lymph nodes, age, alcohol, or tobacco abuse.

**Results:**

There was no differences between private and statutory insured patients concerning overall (*p* = 0.858) or disease-specific survival (*p* = 0.431). Private insured patients received more preoperative PET-CT (*p* = 0.046) and had a better dental status (*p* = 0.006). The occupational groups showed also no differences in survival (*p* = 0.963). The hospitalization of freelancers was in average 2 days shorter. Physically hard workers were diagnosed with bigger tumors (*p* = 0.018) and consumed more tobacco and alcohol. The 5-year survival rate of married patients was approximately 20% points better than not married patients, without showing a significant difference over the entire observation time (*p* = 0.084).

**Conclusion:**

In our cohort, socioeconomic factors have just a limited influence on the survival or treatment of patients with oral cancer. A sufficient statutory health insurance system is a reasonable explanation for this.

## Introduction

With about 275,000 cases a year, the oral and oral/pharyngeal squamous cell carcinoma is the sixth most common tumor in the world, two-thirds of these cases are occurring in the developing countries. However, the mortality from oral cancer had also been rising in first world countries within the last decades [23]. Risk factors for tumorigenesis are mainly tobacco abuse and excess consumption of alcohol [7]. Smoking became less accepted in the western society during the last 40 decades and huge anti-smoking campaigns were implemented to educate the population concerning health issues. It seems to be obvious that those parts of the population who have only limited access to educational opportunities may profit the least from this development. Furthermore, people with less education are often inferior concerning potential earnings. Consequently, they can just afford a basic health insurance or have no health insurance at all, depending on the country they are living in [16, 17]. Today, a lot of first world countries implemented obligatory statutory health insurance systems to cover most parts of their population. However, due to private health insurance which is mainly affordable by higher social classes, there is often the accusation of a two-tier medicine [16, 17]. Critics of this system complain about worse diagnosis and therapy or a longer waiting time for a doctor’s appointment for statutory health insured patients.

Financial possibilities and the educational status are important but they are only two criteria to assess the social status of a patient. In fact, the influence of the social environment on the recovery of a patient should not be underestimated. For example, for a lot of diseases, patients showed a better healing process if they are married. Concerning tumor diseases even survival was increased [3].

This study addresses the following questions in a cohort of patients with oral squamous cell cancer who were treated at the department of oral and maxillofacial surgery at the university hospital of Regensburg Germany: (1) Is there any difference between statutory and private insured patients in diagnosis, influencing factors, and survival? (2) Do the employment and educational status of the patients have an influence on health factors? (3) Do married patients have advantages concerning recovery and survival?

## Material and methods

Of over 1,200 patients with cancer of the oral cavity who were registered at the local tumor centrum between November 2003 and March 2018, we included 400 patients in this retrospective single-center study. Inclusion criteria were the following: all patients were primary cases and underwent an operation of an invasive squamous cell carcinoma of the oral cavity in the department of oral and maxillofacial surgery (university hospital of Regensburg, Germany) in curative intention. Patients were covered by statutory or private health insurance of the German heath care system. Patients with recurrent tumors, unknown primary tumors, tumors of other origin (e.g., salivary glands), and two-stage surgery procedure and patients with incomplete medical records were excluded. The study was approved by the local ethical committee (No. 17–693-104). Clinical data were obtained from patients’ records, the hospital data base system (SAP), and the tumor registries. Pathological TNM classification was recorded according to the guidelines defined by the Union for International Cancer Control (UICC 7^th^ edition).

Basic social and demographic information and risk factors were recorded, including obesity; marital, employment, and insurance status; and alcohol or tobacco abuse.

The occupation of the patients was taken from the anamnesis questionnaire which is included as a standard in our patients’ record and was categorized into 6 groups: (i) unemployed; (ii) worker (physically light work); (iii) worker (physically hard work); (iv) employee (university graduate); (v) freelancer; and (vi) retired persons.

The marital status was also taken from the patient file and divided into 4 groups of persons: (i) single, (ii) married, (iii) divorced, and (iv) widowed.

The health insurance status is classified into the two insurance systems existing in Germany: (i) privately insured and (ii) statutorily insured.

Dental status was evaluated on panoramic radiographs preoperatively. It was distinguished between (i) all supporting zones well preserved (anterior and posterior teeth of the upper and lower well preserved), (ii) one adequate supporting zone well preserved (posterior teeth of the upper and lower jaw of one side well preserved), and (iii) no adequate supporting zone well preserved (no teeth remaining or single teeth with no counterpart remaining).

Length of stay at intensive care and stay at ward including mobilization and administered pain medication was recorded.

Data were analyzed with SPSS for Windows, version 25.0 (SPSS, IBM, Ehningen Germany). Relationships between parameters were examined using the Chi-squared tests (*p* < 0.05) and Fisher’s exact tests (*p* < 0.05) for dichotomized variables. For continuous variables Student’s *t*-test was used if there were two groups to compare. If there were more groups, analysis of variance was used. Overall survival was calculated with the Kaplan–Meier method; distributions were compared by means of the log-rank test. Cox proportional hazards model was used in multivariate analyses. Multivariate logistic regression analysis was conducted to identify independent predictors for overall and disease specific survival (*p* < 0.05).

## Results

**Fig. 1:**
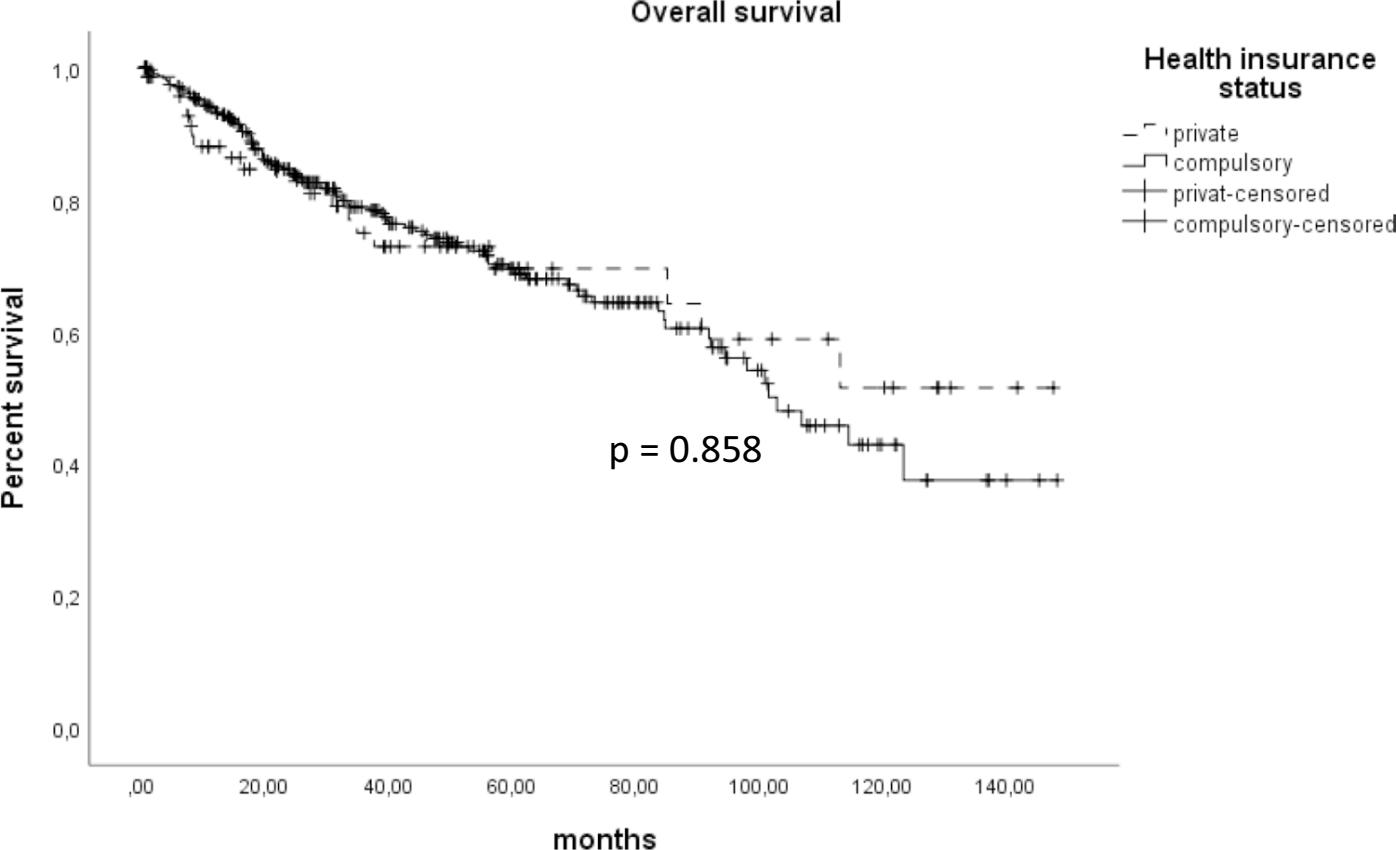
Kaplan Meier for overall survival showed no difference between statutory and private insured patients (*p* = 0.858)

There was no difference concerning overall (*p* = 0.858) (Fig. 1) and disease-specific survival (*p* = 0.431) between private and statutorily insured patients. Private insured patients had a significant better dental status (*p* = 0.006). The waiting time from the first consultation to the operation differed not between the two groups. Statutorily insured patients received the same staging examinations as private insured patients except PET CT scans (*p* = 0.046). No differences between the two groups could be found in terms of tumor size either (*p* = 0.406). Further patients’ characteristics are provided at Table 1.

**Table 1:** Insurance status

Number	Statutory health insurance (*n* = 319)	Private health insurance (*n* = 81)	*p* value	Odds ratio	95% CI
Sex female (vs. male)	101 (218)	34 (47)	0.054	0.640	0.388–1.056
Age < 65 (vs. > 65)	123 (196)	36 (45)	0.200	0.784	0.479–1.284
Waiting time ^a^ (std. dev.)	15.4 (9.4)	15,0 (10,4)	0.244		
Nicotine abuse (%)	186 (58.3)	40 (49.4)	0.093	1.433	0.879–2.338
Alcohol abuse (%)	141 (44.2)	27 (33.3)	**0.049**	1.584	0.949–2.644
Dental status ^b^ (%)	25 (7.9)	15 (18.5)	**0.006**	2.664	1.331–5.330
T1/2 vs.T3/4 (%)	224 (70.7)	59 (72.8)	0.406	1.113	0.645–1.922
N − vs N + (%)	201 (64.0)	56 (69.1)	0.233	1.259	0.745–2.128
Staging					
CT-scan neck (%)	294 (91.8)	70 (87.5)	0.137	1.680	0.771–3.659
CT-scan thorax (%)	144 (45.1)	37 (46.3)	0.478	0.956	0.585–1.564
MRI (%)	62 (19.4)	20 (25.0)	0.171	0.724	0.406–1.289
PET CT scan (%)	101 (31.7)	34 (42.5)	**0.046**	0.627	0.379–1.036

^a^Days (mean) from first consultation to operation

^b^All supporting zones well preserved

There was no difference concerning overall (*p* = 0.963) and disease-specific survival (*p* = 0.789) between the different occupational groups. Retired patients showed a worse outcome due to higher average age (*p* < 0.001). Physically hard workers consumed the most frequent tobacco and alcohol of all occupational groups and furthermore develop bigger tumors (*p* = 0.018) and had the worst dental status. Freelancer had the shortest hospitalization with an average stay of 14.5 days. Physically light workers need the most pain killers within the postoperative phase (Table 2).

**Table 2:** Characteristics of the different occupational groups

Number (%)	Unemployed *n* = 77	Worker (physically light work) *n* = 92	Worker (physically hard work) *n* = 50	Employee (university graduated) *n* = 41	Freelancer *n* = 20	Retired person *n* = 120
Age (average)	62.3	57.2	55.5	60.9	56.3	73.4
Nicotine abuse	62 (80.5)	53 (57.6)	40 (80.0)	17 (41.5)	11 (55.0)	43 (35.8)
Alcohol abuse	43 (55.8)	41 (44.6)	33(66.0)	12(26.3)	10 (50.0)	29 (24.1)
Hospitalization, mean days (std. dev.)	17.2 (8.8)	17.3 (9.5)	19.7 (10.5)	15.3 (7.3)	14.5 (8.6)	17.4 (8.8)
Stay intensive care, mean days, (std. dev.)	3.9 (3.7)	3.6 (3.9)	3.4 (2.2)	2.5 (1.5)	2.8 (2.7)	3.3 (3.4)
Dental status ^a^	8 (10.4)	10 (11.0)	1 (2.0)	11 (26.8)	4 (20.0)	6 (5.0)
T1/2 vs. T3/T4	62 (80.5)	72 (78.3)	28 (57.1)	19 (78.3)	15 (75.0)	94 (64.4)
N0 vs N +	47 (61.8)	64 (69.6)	28 (56.0)	23 (56.1)	15 (78.9)	80(68.3)
Increased pain medication	24 (31.2)	47 (51.1)	17 (34.0)	16 (39.4)	3 (15.0)	48 (40.0)
Tranquilizer	10 (13.0)	17 (18.5)	4 (8.0)	9 (22.0)	2 (10.0)	12 (10.0)
Insurance status: private vs. statutory	12 (15.6)	9 (9.8)	0 (0.0)	33 (80.5)	9 (45.0)	18 (15.0)

^a^All supporting zones well preserved

**Fig. 2:**
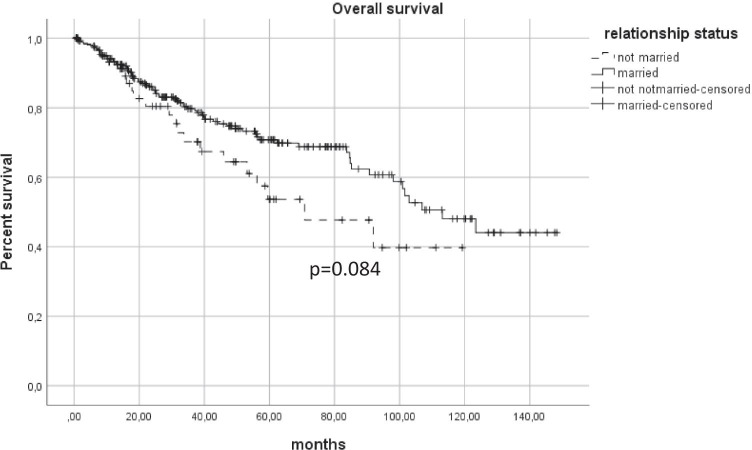
Kaplan Meier for overall survival showed no difference between married and not married patients. Married patients had a better 5-year survival rate (70.8%) than not married (single, divorced, widowed) patients (53.7%)

Although there was no difference concerning overall survival (*p* = 0.084) (Fig. 2), married patients had a better 5-year survival rate (70.8%) than not married (single, divorced, widowed) patients (53.7%). Subgroup analysis of single, divorced, or widowed patients showed also no difference. Potential influencing factors like age (*p* = 0.086), tumor size (*p* = 0.370), positive nodal status (*p* = 0.097), nicotine (*p* = 0.189), and alcohol abuse (*p* = 0.059) varied not between married and not married patients. Length of stay and the mobilization process were not influenced by marital status. Besides, patients with T3 and T4 tumors (*p* = 0.001) and patients with a BMI under 18.5 (*p* < 0.001) had a prolonged mobilization process, whereas obese patients had no delay in mobilization compared to normal weight patients (BMI: 18.5–25).

Independent risk factors for a worse overall or disease-specific survival were tumor size, positive lymph nodes, and age. The analyzed socioeconomic factors had no influence on survival (Table 3).

**Table 3:** Cox regression multivariate analysis

	Overall survival				Tumor-specific survival			
	Hazard ratio	CI 95%		*p*-value	Hazard ratio	CI 95%		*p*-value
		Lower	Upper			Lower	Upper	
Married patients	0.872	0.578	1.315	0.513	0.466	0.112	1.937	0.294
Nicotine abuse	1.064	0.714	1.586	0.760	0.853	0.532	1.367	0.508
Alcohol abuse	1.060	0.727	1.546	0.761	0.967	0.599	1.562	0.892
Age over 65	2.114	1.394	3.206	** < 0.001**	2.262	1.414	3.619	**0.001**
Tumor size	1.878	1.257	2.805	**0.002**	2.103	1.266	3.492	**0.004**
Nodal positive	2.098	1.426	3.089	** < 0.001**	1.942	1.191	3.166	**0.008**
Occupation “hard worker”	0.860	0.422	1.752	0.679	0.744	0.317	1.748	0.498
Private insurance	1.105	0.677	1.805	0.689	0.833	0.472	1.470	0.529

## Discussion

Although there are complex indices to describe occupational burden [18], it is difficult to determine socioeconomic and educational factors in their entirety and their attribute on the outcome of a serve disease. By focusing on occupation/education and marital and insurance status, we evaluate socioeconomic factors which were mainly discussed in the literature as most influencing on the outcome of cancer diseases. However, most studies just evaluate just one of these factors [1, 3, 8, 18].

It is a matter of course that patients’ work-life has an influence on their behavior concerning heath issues. For example, we observed that the average hospital stay of freelancers was 3 days shorter in comparison to the other occupational groups. This phenomenon is typical for non-paid sick leave. Suspecting reduced earnings, self-employed patients aspire an early discharge and often start working before full recovery. Non-paid sick leave employees are also afraid of job loss ahead of the financial loss; consequently, they are more likely to come to work when they are sick [19]. Besides, they tend to forgo needed medical care [2]. If the awareness of a severe illness is also reduced under those circumstances is open to speculation. But in our cohort, there is no indication for late diagnose or bigger tumors for freelancers.

The occupational group of hard workers consumed the most alcohol and tabaco and also had significant more T3 and T4 tumors. Interestingly, this unfavorable condition did not result in a worse survival rate. A reasonable explanation might be that this group was the youngest, with an average age of 55 (*p* < 0.001). Considering this, they are also the occupational group which develops their tumors first. Additionally, this group had the worst dental status and no patients were covered by private insurance. In contrast, university graduate patients profit from the best dental status and were covered the most with private health insurance of all occupational groups. However, this results not in a significant advantage referring to overall or disease-specific survival. A lot of studies showed a correlation between not well-educated classes of population and the risk of developing a cancer disease or a poor outcome after treatment [1, 9, 18]. However, especially for oral cancer, there are also well-conduced epidemiological studies which were not able to demonstrate a correlation between survival and level of education [24]. Unfortunately, for this entity, race plays also an important role. A study of the University of Pittsburgh showed that even after adjusting to socioeconomic and insurance status and matching to influencing risk factor like tobacco or alcohol, African-Americans were more likely to be diagnosed with advanced oral cancer stages compared to white patients [15]. In our cohort, we are not able to address this topic because there is no documentation of ethnical origin as a standard in our records.

Independent of the occupational group, the status of insurance was less important than expected. Private insured patients received more preoperative diagnostics by PET-CT scan, but there was no difference in waiting time for the operation appointment, and more importantly, there was also no correlation to late cancer stage. Although PET-CT scans are able to accurately detect tumor formations, there was no advantage concerning tumor outcome. Besides, PET-CT scans are also not recommended for the initial staging in the German guidelines for oral cancer treatment. They are more important for the diagnostics of recurrence tumors. CT scan or MRI is sufficient and the preferred diagnostic procedures at the staging of primary cases. In our cohort, private insurance privileges were presented by a sufficient dental status. A reasonable explanation is that the German statutory health only covers a small part of the costs of a dental prosthesis. Hence, the high additional costs are hardly affordable for the average statutory insured patient. Alcohol abuse was more likely to be in the statutory insurance group, but it was no independent factor for survival influence. Consequently, the survival outcome differs not between private and statutorily insured patients. But one should always keep in mind that our results have to be seen in the context of an advanced developed country. Therefore, the socioeconomic range might be less and have limited influence on the outcome of the treatment of a specific disease.

A lot of studies showed advantages for private insured patients. Head and neck cancer patients who were treated at the University of Pittsburgh Medical Center had a worse survival outcome if there were uninsured or covered with Medicaid/Medicare compared to private insured patients. Furthermore, there were more likely to present with an advanced tumor stage or positive lymph nodes [6]. Additionally, the outcome was worse independent of cancer stage, alcohol use, smoking, gender, age, and race. Similar results showed a recent study of Panth et al. who examined a cohort of 18,923 female who were diagnosed with head and neck cancer between 2007 and 2014 in the USA [13].

However, variable treatment outcomes resulting on health insurance status have to be seen on the basis of the healthcare systems in the different countries. However, multiple barriers are responsible for an aggravated access to healthcare: high costs of the healthcare system in general, lack of transportation possibilities to medical appointments, or due to financial stress in which people are avoiding doctors’ appointments so they will not be absent at work. In the USA, people with basic health insurance are often confronted with high additional cost, if a complex therapy is needed. Especially for head and neck cancer therapy, the possible treatment costs are approximately 40,000 USD for radio-chemotherapy (surgery not included) [11] and therefore only hardly affordable for the average citizen. In contrast, a statutory insurance, which covers the full needed treatment and paid a sick leave, guarantees the financial support of the patient during recovery. This might explain the missing advantages of a private insured patient in a country with a long tradition of statutory health care. At least for this cohort, there is no indication for a preferential treatment of private insured patients, which enfeebles the criticism of a two-tier medicine.

Our results indicate that married patients had a better outcome referring to survival. Five years after diagnosis, the overall survival rate was approximately 20% higher for married patients than for not married patients. However, the survival curves converge at the following observation period, leading to a not significant different outcome (*p* = 0.084) (Fig. 2). The importance of social support during and after cancer disease is well reported for a lot of entities. Osborne et al. showed in his breast cancer study that older not married patients are more likely to be diagnosed late and had worse survivals rates [12]. However, in our cohort, married patients had no advantage concerning tumor size or nodal status compared to not married patients. Consequently, the assumption that not married patients are more isolated and have less awareness of their disease leading to a later diagnosis did not apply to our cohort. Concerning cancer survival, a lot of studies emphasized the importance of a perceived social support and social network including the marital status [14]. On univariate analysis, Eskander et al. showed a longer survival for breast, lung, colorectal, kidney, and pancreatic cancer. But only at patients with lung and breast cancer marriage serve as an independent predictor for improved survival [3].

Serval explanations were discussed why a better social network positively affects the mortality of cancer diseases. A fully functional social life makes it easier for patients to get support in care, keep their medication compliance and doctors’ appointments [5, 14]. Furthermore, the risk of a post-interventional depression, which causes a higher mortality itself, is decreased, if patients are socially supported [20, 22]. Although there are indications that married patients have an improved functional recovery after certain surgery [4, 10], in our study, the average length of stay in hospital was the same. Length of stay was only prolonged if patients had bigger tumors or were underweight independent of their marital status.

There are serval limitations of this study. Although occupational status has the advantage of being easily collected, it only reflects a snapshot at the time of diagnosis. Our data collection makes no differentiation between a well-educated unemployed patient who is just between two jobs or a longtime unemployed person. The same problem occurred in the group of retired persons. Here, patients indicated themselves as a retired person independent of their former job. Furthermore, the categorization into 6 occupational groups has to be a subjective procedure. For example, a mason was grouped at “physically hard worker” and an office worker was grouped at “physically light worker.” However, we are aware that physically hard work could also be part of an office job.

Similar to the occupational status, the marital status has to be seen in context with the time of diagnosis. Any change of marital status after the treatment was not recorded. Additionally, it is obvious that a supportive social surrounding is not exclusive for married patients. Persons in long-term cohabitation may have the same support than happily married persons. We were not able to distinguish between these relationships and true singles.

Although patients were examined for general disease due to anesthesiologic reasons before the operation, they were not checked as a standard for diseases like HIV and hepatitis B or C. These infections might be confounding variables influencing the outcome of a cancer disease. Especially, hepatitis B is known for increasing the risk for developing many cancer types including oral cancer [21].

## Conclusion

The influence of socioeconomic factors on the outcome of oral cancer patients is limited in our cohort. There were some indications for a better overall survival for married patients after 5 years, but the differences were not significant over the whole observation period. However, social support should not be underestimated during recovery of a sever disease. Similarly, the occupational groups showed only slight differences. In fact, well-educated patients were more likely to be privately insured. But there was no advantage concerning education or insurance status, neither for survival in general nor assumed privileges like waiting time. Sole exception was more diagnostic preoperative examination with PET CT scan at the private insured group, which also had no influence on patients’ outcome. However, privately insured patients profit from a better dental status. Our study illustrates that the well-established statutory insurance system in Germany is sufficient to cover a state-of-the-art treatment of a specific disease. Especially for potential lethal diseases, social disparities should not be the crucial factor for the outcome.
